# A Linearity-Enhanced Time-Domain CMOS Thermostat with Process-Variation Calibration

**DOI:** 10.3390/s141018784

**Published:** 2014-10-10

**Authors:** Chun-Chi Chen, Yi Lin

**Affiliations:** Department of Electronic Engineering, National Kaohsiung First University of Science and Technology, Kaohsiung 811, Taiwan; E-Mail: u0252807@nkfust.edu.tw

**Keywords:** thermostat, CMOS, time-domain, linearity enhancement

## Abstract

This study proposes a linearity-enhanced time-domain complementary metal-oxide semiconductor (CMOS) thermostat with process-variation calibration for improving the accuracy, expanding the operating temperature range, and reducing test costs. For sensing temperatures in the time domain, the large characteristic curve of a CMOS inverter markedly affects the accuracy, particularly when the operating temperature range is increased. To enhance the on-chip linearity, this study proposes a novel temperature-sensing cell comprising a simple buffer and a buffer with a thermal-compensation circuit to achieve a linearised delay. Thus, a linearity-enhanced oscillator consisting of these cells can generate an oscillation period with high linearity. To achieve one-point calibration support, an adjustable-gain time stretcher and calibration circuit were adopted for the process-variation calibration. The programmable temperature set point was determined using a reference clock and a second (identical) adjustable-gain time stretcher. A delay-time comparator with a built-in customised hysteresis circuit was used to perform a time comparison to obtain an appropriate response. Based on the proposed design, a thermostat with a small area of 0.067 mm^2^ was fabricated using a TSMC 0.35-μm 2P4M CMOS process, and a robust resolution of 0.05 °C and dissipation of 25 μW were achieved at a sample rate of 10 samples/s. An inaccuracy of −0.35 °C to 1.35 °C was achieved after one-point calibration at temperatures ranging from −40 °C to 120 °C. Compared with existing thermostats, the proposed thermostat substantially improves the circuit area, accuracy, operating temperature range, and test costs.

## 1. Introduction

With the rapid increase in the circuit density and clock speed of modern very large-scale integration (VLSI) systems, all electronic circuits and systems can be affected by problems caused by heating, which severely affects their reliability. In the absence of appropriate safeguards against heating, components are susceptible to damage when the temperature exceeds their safe operating range, invariably resulting in system failure. A thermostat is a critical component of a thermal management system, and it is used to monitor and maintain the temperature of a system, room, or building. Thermostats are widely used in many industrial applications and have been gradually integrated into homes, thereby increasing the market demand for thermostats. To facilitate cost-effectiveness and the direct monitoring of temperature, thermostats are typically mounted near crucial heat sources. Thus, for the purpose of on-chip monitoring, integrated thermostats can be implemented using complementary metal-oxide semiconductor (CMOS) technology to enable simple integration with other VLSI circuits to maintain temperature conditions by managing the temperature.

To indicate the relationship between the temperature and a preset or user-programmable temperature set point, the thermostats must have a logic-level output. A temperature sensor is used to sense the ambient temperature and generate the temperature-dependent voltage signal *V_D_*. By using external resistors, a capacitor network, or a digital-to-analogue converter [1–4], the thermostat can determine the adjusted set-point voltage *V_A_*. For example, the resistor values required for achieving a desired temperature set point are calculated using a universal formula. When *V_D_* is greater than *V_A_*, an output signal informs the thermal management system that either the fan speed should be increased or the operation frequency should be decreased to avoid thermal damage. A crucial feature of thermostats is that they show hysteresis in the desired range to prevent controlled equipment from switching too frequently when a detected temperature is at (or near) the programmed set-point temperature.

**Figure 1.**
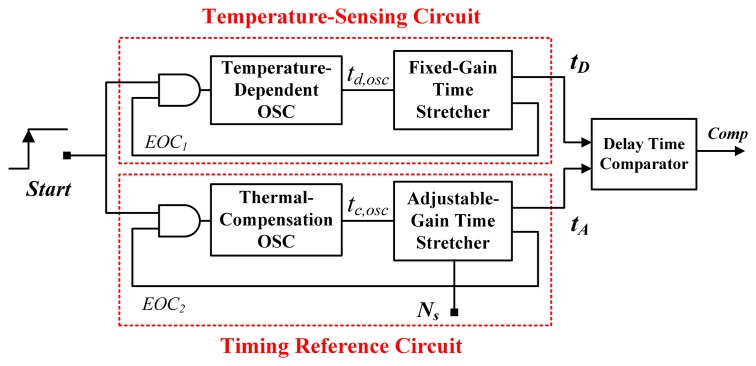
Block diagram of former time-domain thermostat [6].

A previous study proposed that an inverter-based thermostat with time-domain operation is suitable for programmable set-point operation [5]. The study also showed that inverter-based thermostats require considerably less circuit area and are less complex compared with voltage-domain thermostats. Figure 1 shows a block diagram of an existing time-domain thermostat [6]. It consists of a temperature-sensing circuit for generating a temperature-dependent time *t_D_*, a timing reference circuit for determining an adjustable set-point time *t_A_*, and a delay-time comparator for detecting the relationship between *t_D_* and *t_A_*. This architecture is advantageous because it requires no external components, and it allows the use of nearly-all-digital circuits, thereby enabling integration with other VLSI systems. However, it also exhibits the poorer linearity because of the curvature caused by the temperature-dependent circuit [5–8]. To improve accuracy, the curvature compensation using two delay lines was devised, yielding the high accuracy of −0.35 to 0.3 °C over a 0 to 90 °C range [7]. Furthermore, to increase the temperature range operability and to ensure the acceptable inaccuracy, a more effective curvature compensation technique using two oscillators was invented, yielding a maximum inaccuracy of only 1.4 °C and widening the operating temperature range from −40 to 120 °C [8]. A previous study proposed a cross-coupled-structure-based temperature sensor with two oscillators [9]. The errors and the sensitivity of process variations can be reduced because of the compensation between the two oscillators. A simulated error of ±1.1 °C in a typical corner was achieved between −40 °C and 120 °C. In addition, the inaccuracies of these time-domain sensors were resolved by using two-point calibration to compensate for the sensor's sensitivity to process variations [5–9]. Compared with two-point calibration, one-point calibration halves the test cost. Thus, the test cost of high-volume production is higher than that of voltage-domain sensors that achieve acceptable inaccuracy by using one-point calibration and voltage-domain curvature correction techniques [10,11].

**Figure 2.**
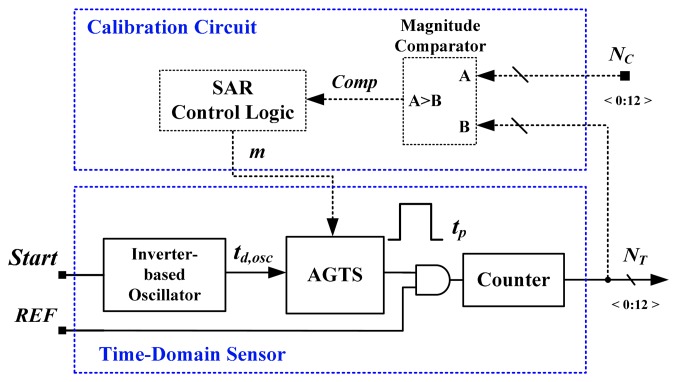
Basic architecture of a time-domain sensor with one-point calibration support [13].

To reduce the cost of tests, numerous previous studies have proposed using time-domain sensors with one-point calibration [12–18]. In [12], an inverter-based temperature sensor with a cell-based design was presented. The proposed device mitigated the effect of process variations by performing self-calibration with a reference clock. An inaccuracy of −5.1 to 3.4 °C was achieved in the range of 0–60 °C after one-point calibration. The core area was 0.01 mm^2^ in a 65-nm CMOS process, and power consumption was 150 μW at 10k samples/s. Another all-digital version that is realizable with field programmable gate array (FPGA) logic was proposed [13]. The basic architecture of the time-domain sensor equipped with an off-chip calibration circuit is shown in Figure 2. The process-variations calibration technique is similar to that used in this study. An inverter-based oscillator for temperature sensing was used to generate the oscillation period *t_d,osc_* proportional to absolute temperature (PTAT). The adjustable-gain time stretcher (AGTS) was adopted to compensate dynamically for *t_d,osc_* to mitigate the effect of process variations; a calibration circuit comprising a magnitude comparator and successive-approximation-algorithm (SAR) control logic, was used with the AGTS. Only 48 logic elements were realized and 175 μW at 1 k samples/s was consumed. However, as mentioned, the characteristic curve of the CMOS is large, which severely limits the accuracy and operating temperature range. For accuracy improvement, an off-chip second-order master curve-fitting process was adopted to reduce the curvature, and the error was reduced to −0.7 to 0.6 °C from 0 to 100 °C. However, the fitting procedure increased the test cost and time. With the similar calibration technique, a 0.13-μm delay locked loops (DLLs)-based CMOS temperature sensor was created [14]. Two DLLs were used to successfully solve the problem caused by the process variations of inverter-based sensors. The measurement errors reached an error of −4 to 4 °C within a 0–100 °C range. The large circuit area and power consumption were consumed because of the DLL-based structure.

Without adopting the fitting for curvature correction, a frequency-to-digital-based temperature sensor using a multiphase clock was proposed to achieve a little bit large inaccuracy of −2.8 to 2.9 °C after one-point calibration from −40 to 110 °C [15]. The sensor, which exhibited an area of 0.0066 mm^2^ in a 65-nm CMOS technology, featured a high conversion rate of 366 kHz and a power consumption of 400 μW. A process-variation-calibrated temperature sensor was also proposed for one-point calibration support [16]. When using the off-chip curve fitting, an acceptable inaccuracy of −0.6 to 1.0 °C form 20 to 120 °C was obtained. The circuit, which featured a low energy consumption of 289 μW at a 430 kHz conversion rate, exhibited an area of 0.031 mm^2^ in a 0.13-μm CMOS process. An oscillator-based self-calibrated temperature sensor with an on-chip process compensation circuit was proposed in [17]. By using a TSMC 65-nm CMOS process, the sensor required a layout area of only 0.0015 mm^2^ and achieved a simulated inaccuracy of −1.5 °C to 1.3 °C at temperatures from 0 °C to 130 °C. Without increasing the circuit overhead, a previous study proposed a continuous self-calibration technique for removing process variations [18]. The all-digital sensor with an on-chip self-calibration circuit was implemented on 65-nm FPGAs and had 60 logic elements. However, the sensor achieved an error of ±1.6 °C at operating temperatures of 20 °C to 75 °C only. An online model with performance counters was proposed in [19] to estimate the temperature of multiple sensor locations on a silicon die. A novel algorithm was used to correct the temperature readings, and an average error of 1.5 °C was achieved. In [20], the cyclic dependence between leakage power and temperature was modelled to evaluate the calibration accuracy of the sensor. The two calibration techniques [19,20] were presented to directly calibrate the on-chip temperature sensors to effectively reduce the test time and cost.

To overcome the curvature and test-cost problems, an on-chip linearity-enhanced technique is proposed in this study. The AGTS is adopted to perform one-point calibration for test cost reduction. The proposed thermostat yields acceptable inaccuracy following one-point calibration at operating temperatures between −40 °C and 120 °C. The remainder of this paper is arranged as follows: Section 2 details the circuits of the proposed thermostat, including a new linearity-enhanced temperature-sensing cell. The measurement results are presented in Section 3, and finally, Section 4 offers the conclusion.

## 2. Circuit Description

**Figure 3.**
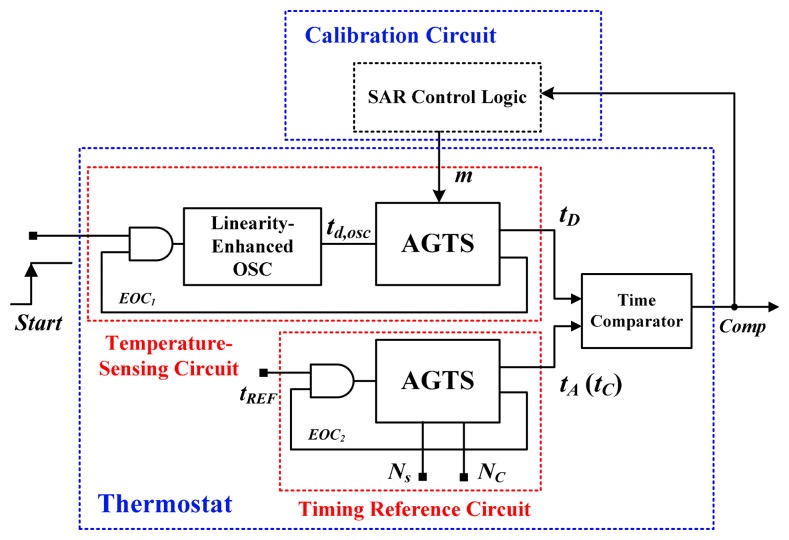
Block diagram of the proposed time-domain thermostat.

Figure 3 shows the architecture of the proposed time-domain thermostat, which is similar to architecture reported in [5,6], and it comprises a temperature-sensing circuit, a timing reference circuit, a delay-time comparator, and a SAR control logic. The proposed thermostat differs from those proposed in previous studies because it features a linearity-enhanced temperature-dependent oscillator [5,6] and uses SAR logic for the calibration circuit [13]. Additionally, the costly timing reference delay line [5] and oscillator [6] are replaced with a simple reference clock.

First, the highly linear *t_d,osc_* PTAT was produced by the temperature-dependent oscillator. The AGTS with an input code *m* controlled by the SAR logic was then used to dynamically adjust the time gain for the linearized period so as to generate the temperature-sensing delay *t_D_* with process-variation calibration. The programmable adjusted delay *t_A_* controlled by the digital set-point code (*N_s_*) was generated by the reference clock *t_REF_* and another AGTS identical to the aforementioned AGTS. The parameter *t_A_* is an excellent timing reference delay because *t_REF_* is process-, voltage-, and temperature-insensitive. Although the two AGTSs perform distinct functions, they are identical, thereby reducing the complexity of the circuit design. When the two delays were generated, end-of-conversion (EOC) signals were initiated to shut down the oscillator to reduce the power consumption. A delay-time comparator was adopted to detect the timing difference between *t_D_* and *t_A_*, and to generate *Comp* either for the SAR logic to adjust the time gain in the calibration mode, or for the thermal management system to control the temperature in the measurement mode.

In the calibration mode, the calibrated value *N_C_* denotes a preset value at the calibration temperature *T_C_* for process-variation calibration. Regarding the measurement mode, *N_s_* was used to determine the programmable temperature set point. Compared with previous versions of the two-point calibration [5–9], satisfactory accuracy can be achieved after one-point calibration in the wide temperature range of −40 °C to 120 °C because the linearity enhancement and process-variation calibration effectively reduce the test cost of high-volume production while enabling a wider range of operating temperatures.

### 2.1. Proposed Linearity Enhancement for the CMOS Inverter-Based Buffer

The CMOS inverter can be regarded as a simple PTAT delay cell that is used to sense temperatures [5–9,12–18]. The invertor's temperature-dependent propagation delay *t_inv_*(*T*) was theoretically derived in [7,8,12], and can be written as shown in [21]:

(1) $$t_{inv}\left(T\right)=\frac{2LC_{L}T_{0}^{km}}{\mu_{0}WC_{OX}V_{DD}}\times \frac{\ln\left(3-4V_{th}/V_{DD}\right)}{1-V_{th}/V_{DD}}\times \frac{1}{T^{km}}=\gamma \times T^{-km}$$

where *μ_0_*, *T*, *T_0_*, *V_th_*, *W/L,* and *C_L_* are reference carrier mobility, operation temperature, reference temperature, threshold voltage, effective aspect ratio of transistors and loading capacitance of the NOT gates. And *γ* is a process-dependent constant that is nearly independent of the temperature. The exponent −*km* of temperature *T* is the parameter of carrier mobility (*μ*) and is considered independent of the temperature [22]. In (1), *km* dominates the temperature-dependent term and its value ranges from −1.2 to −2.0. The characteristic curve of the inverter shows some curvature because the value of −*km* is not equal to 1. The larger the deviation between −*km* and 1, the larger the curvature of *t_inv_*(*T*) and the higher the corresponding inaccuracy [8]. In other words, the wider the temperature range, the greater the inaccuracy because of the large curvature. For TSMC 0.35-μm CMOS process, the inaccuracy for automobile ICs with the operating range of −40 to 120 °C is four times higher than the inaccuracy for commercial ICs with operating range of 0 to 80 °C [8]. Therefore, when the curvature is reduced, the inaccuracy at wider temperature ranges is improved considerably.

**Figure 4.**
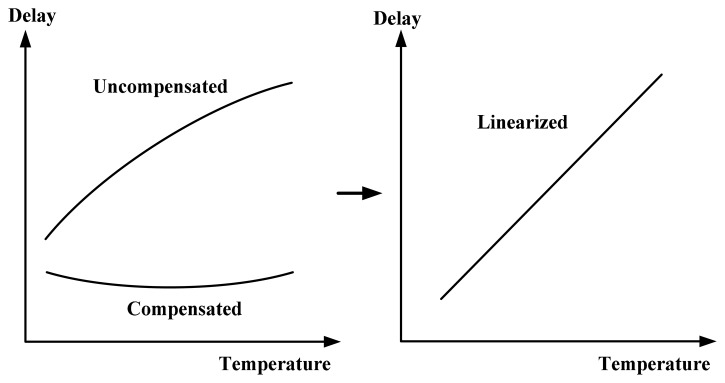
Linearity-enhanced temperature-sensing cell.

To improve the accuracy and increase the operating temperature range, the proposed linearity-enhanced temperature-sensing cell was used. Figure 4 shows the cell circuit, where L denotes a linearised cell. A buffer (*i.e.*, two inverters in series) with a thermal-compensation circuit was added in parallel to the simple buffer to form a new cell to generate the linearised delay PTAT.

**Figure 5.**
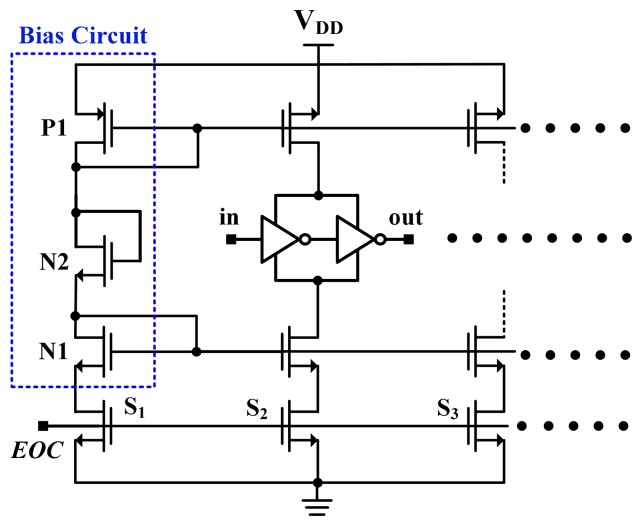
Concept of proposed linearity enhancement.

The concept of the linearity-enhanced technique is presented in Figure 5. The simple buffer exhibits poor linearity because of the large curvature of the buffer. With the proposed linearity enhancement, the compensated delay was designed to produce a slight concave curve to eliminate the convex curve corresponding to the uncompensated delay. Thus, high linearity can be achieved for the combined delay. The linearity-enhanced mechanism differs from the former versions containing two delay lines [7] or two oscillators [8,9] that cannot perform effectively in conjunction with a calibration circuit used for one-point calibration support. Although the effect of the process variations exists obviously for the proposed mechanism, process-variation calibration was adopted to perform a one-point calibration to reduce the test cost and achieve satisfactory accuracy at operating temperatures similar to that of automobiles.

**Figure 6.**
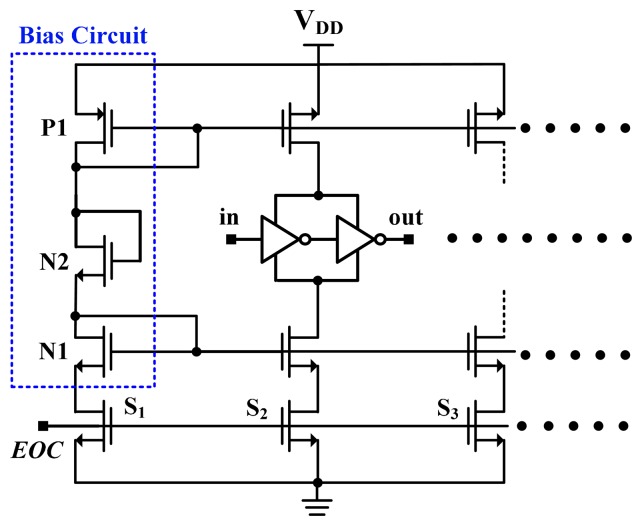
Temperature-compensation circuit for linearity enhancement.

Figure 6 shows the linearity-enhanced thermal-compensation circuit, which is based on the compensation circuit proposed in [8]. The operating principle for thermal compensation was discussed [8]. A bias circuit was designed for the compensated buffer to compensate for the curvature of the uncompensated delay and to achieve a high linear delay. With the assistance of HSPICE simulation, the size of transistors N2, P1, and N1 can be appropriately determined to obtain the desired linearity-enhanced curve. In this study, the compensated buffers shared the bias circuit to reduce the circuit cost. To turn off the compensation circuit and conserve power, the EOC signal was used to control switches.

**Figure 7.**
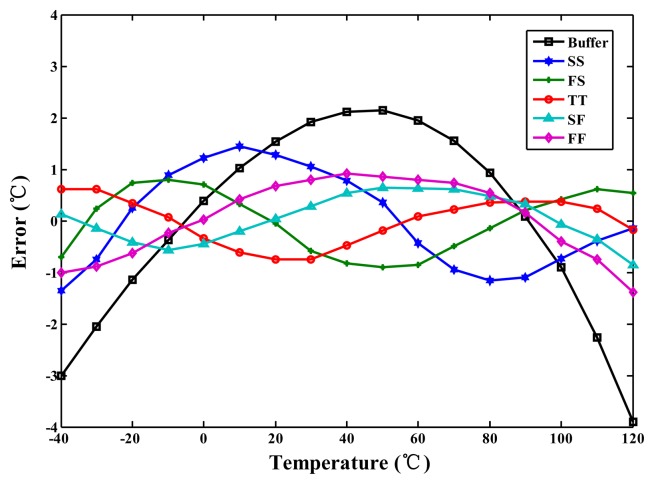
Simulated errors for CMOS buffer and the cell with five-process corners.

**Figure 8.**
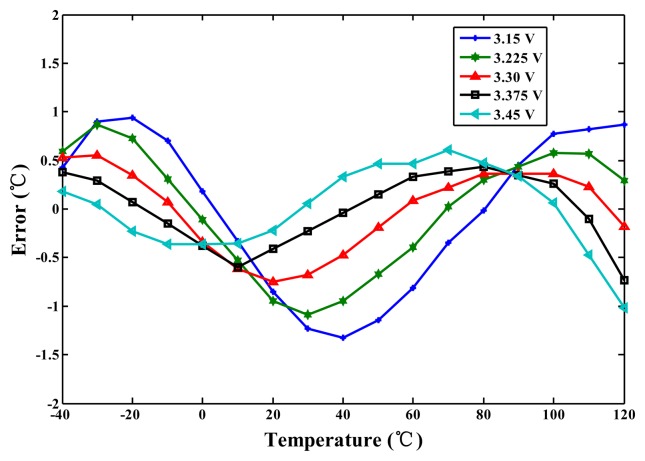
Simulated errors for the cell with voltage variations ±5%.

For the TSMC 0.35-μm CMOS process, the linearised delays associated with the five-process corners (the cell) and the uncompensated delay (CMOS buffer) were simulated for comparison to demonstrate the proposed linearity enhancement. The integral non-linearity (INL) errors observed when the linearity enhancement was and was not applied are shown in Figure 7. For a nominal 3.3 V and TT process, the simulated inaccuracy when the linearity enhancement was applied improved considerably from −3.8 to 2.1 °C to −0.8 to 0.6 °C in the range of −40 to 120 °C. Thus, the accuracy was improved more than four-fold. However, the lowest accuracy (occurring at SS) was −1.3 °C to 1.4 °C, corresponding to a two-fold improvement. For supply voltage variations of ±5%, the simulation results for the cell are presented in Figure 8. The lowest accuracy (occurring at 3.15 V) was −1.4 to 0.9 °C, which is still far superior to the accuracy of the un-linearized version. Thus, the proposed technique for voltage variations is effective.

### 2.2. Temperature-Sensing Circuit with Process-Variation Calibration

**Figure 9.**
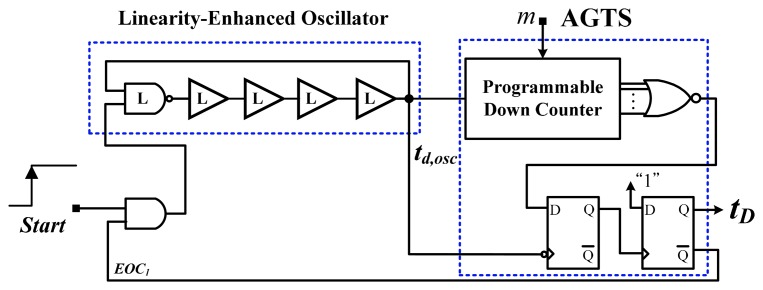
Temperature-sensing circuit.

The temperature-sensing circuit is shown in Figure 9. Because of the successful linearity enhancement of the cell, the number of stages used in the oscillator is related only to the oscillatory period. A wider period reduces the power consumption and decreases the accuracy of the calibration because a larger deviation of the period may exist after the calibration. Consequently, it is compromised in selecting the number of stages for the oscillator. The linearity-enhanced oscillator consists of four buffer-based cells and a NAND-gate-based cell to generate the linearised delay *t_d,osc_* PTAT. The AGTS primarily comprises a programmable down counter and D type Flip Flops (DFFs) to amplify *t_d,osc_* dynamically. The characteristic curve of *t_d,osc_* resembles that of the linearised cell, and thus, after the time amplification, a longer *t_D_* with high linearity can be achieved. In other words, the thermostat can be operated over a wider temperature range, and an acceptable level of accuracy can be achieved following one-point calibration without requiring a costly second-order master curve-fitting.

### 2.3. Timing Reference Circuit for Temperature Set Point

To program the set-point reference delay *t_A_*, *t_REF_* was used along with the AGTS, which was used as a programmable timing generator. The time resolution is the oscillation period of *t_REF_* and it is not expected to be fine because an adequately wide unit delay should be implemented for *t_A_* to markedly reduce the influence of the cell mismatch. Thus, a low frequency can be used to mitigate the power dissipation, and the corresponding period is sufficiently long to overcome the delay variation along various signal paths. Additionally, the larger *t_REF_* (*i.e.*, a wider unit delay) can assist in reducing the effect of the process variations in the calibration mode, thereby improving the accuracy of the calibration. However, this increases the conversion time. A larger *N_s_* results in a later rising time of *t_REF_* being tapped out and a longer *t_A_*. Because *t_A_* is insensitive to the process, voltage, and temperature, one-point calibration can be performed. This study differs from the previous studies [5–8] in that the thermal-compensation delays in previous studies have been insensitive only to temperature. Additionally, the two AGTSs were designed to have the same number of bits to minimize the timing mismatch. The AGTS in the temperature-sensing circuit was adopted for time amplification and process-variation calibration. By contrast, the AGTS in the timing reference circuit was used to derive the adjustable reference delay for the temperature set point.

### 2.4. Calibration Circuit for One-Point Calibration Support

**Figure 10.**
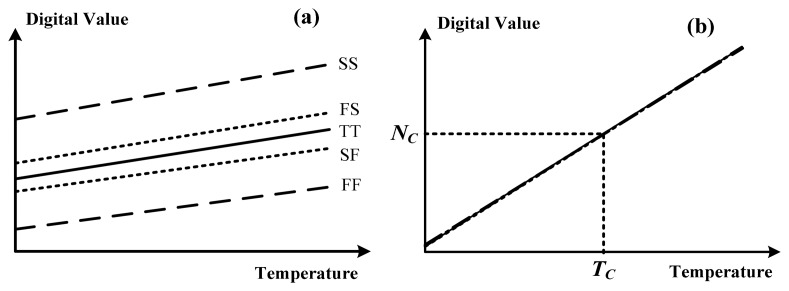
The transfer curves (**a**) without and (**b**) with the calibration technique for five-process corner variations.

As described, the calibration technique used in this study is similar to that used in [13], in which the theory and corresponding operation of one-point calibration support were discussed. To facilitate a comparison, Figure 10 shows the transfer curves without and with using the calibration technique for five-process corner variations. Without the calibration, the digital values *versus* the temperature for the process variations were spread considerably. With the calibration, the values of the process variations nearly coincided at *T_C_* and varied linearly with the temperature. The fluctuation of the values at the same test temperature was improved significantly, thus validating the effectiveness of one-point calibration.

**Figure 11.**
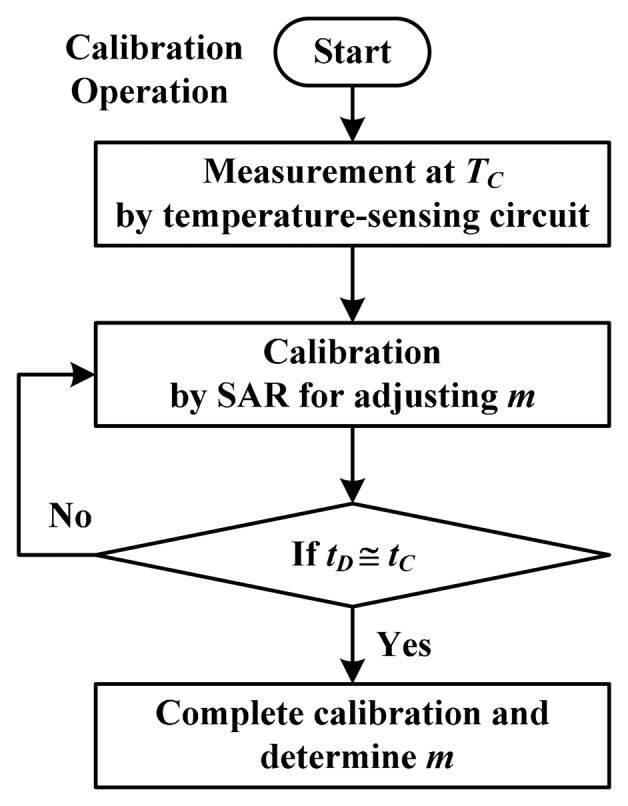
The flowchart for the calibration.

In this study, only SAR logic was adopted for the calibration circuit to assist the process-variation calibration, and it was implemented off-chip to reduce cost, because it is useless after calibrating each thermostat. The magnitude comparator used in [13] was not used because the two delays (*t_D_* and *t_C_*) were directly detected by the delay-time comparator. Figure 11 depicts a flowchart of the calibration. When the calibration procedure was initiated, the temperature of the *i*th thermostat was controlled at *T_C_*. The delay *t_D_* was measured and then dynamically varied by using SAR logic. Finally, the parameter *t_D_* approximated the preset set-point delay *t_C_*, which was determined based on *N_C_* and *t_REF_*, following the adjustment of the corresponding time gain *m_i_* for *t_d,osc,i_*. This calibration condition can be expressed as:

(2) $$t_{C}=N_{C}\times t_{\text{REF}}=m_{i}\times t_{d,\text{osc},i}\left(T_{C}\right)=t_{D}\left(T_{C}\right)$$

The calibration is then completed and *m_i_* for thermostat *i* can be determined as:

(3) $$m_{i}=\frac{t_{\text{REF}}\times N_{C}}{t_{d,\text{osc},i}\left(T_{C}\right)}$$

In this situation, the variation in *t_d,osc_* for the process variations at *T_C_* was compensated for dynamically to generate the same delay, which is equal to *t_C_*. The offset and gain calibrations were both completed. Thus, a fixed temperature resolution *R* was achieved, and it was estimated as:

(4) $$R=\frac{T_{C}-\left(-273.15\right)}{N_{C}}$$

For example, if *T_C_* was set to 40 °C (middle temperature from −40 °C to 120 °C) and the value of *N_C_* was set at 6263, then the resolution would be 0.05 °C.

When entering measurement mode, *N_s_* of the *i*th thermostat regarding the trip temperature can be expressed as:

(5) $$N_{s}=\frac{m_{i}\times t_{d,\text{osc},i}\left(T\right)}{t_{\text{REF}}}$$

**Figure 12.**
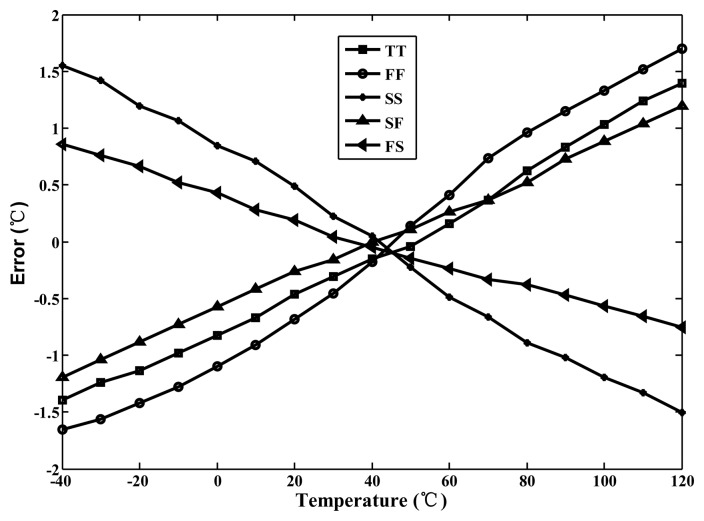
Simulated inaccuracies using the calibration for five process corners.

Because *R* is fixed and all thermostats have the same set-point values at *T_C_*, all calibrated thermostats have similar set-point values at any given trip temperature; therefore, one-point calibration can be performed effectively. To demonstrate the calibration performance of process variations, a simulation was performed to calibrate five process corners, where *N_C_* = 6263 at *T_C_* = 40 °C, and a 14-bit circuit was designed to cover the full operating range of automobile ICs. Figure 12 shows the simulated inaccuracy after one-point calibration. The inaccuracies at 40 °C are −0.2 °C–0.05 °C and those between −40 °C to 120 °C are ±1.7 °C. This validates the function of one-point calibration support.

### 2.5. Time Comparator with Built-In Hysteresis

**Figure 13.**
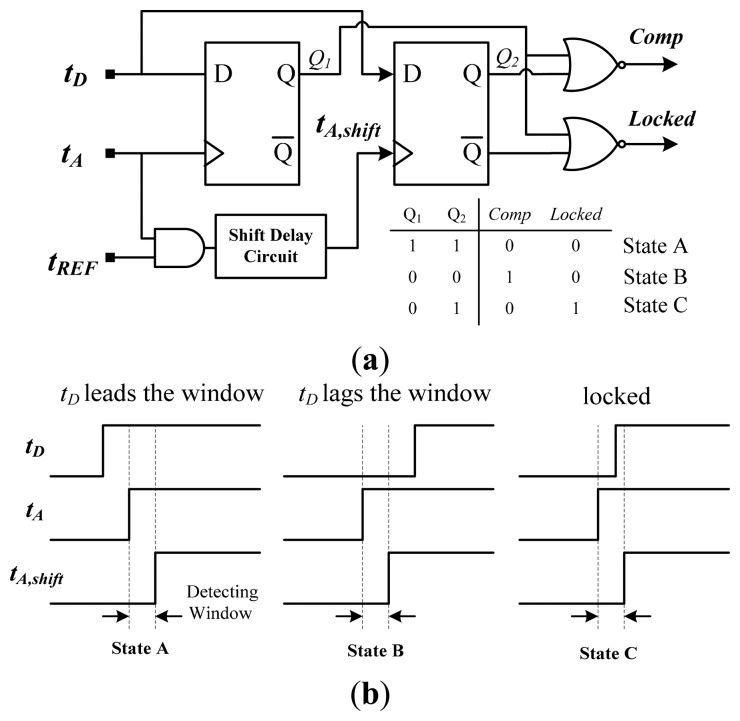
(**a**) Schematic diagram of the delay-time comparator with build-in hysteresis; (**b**) Timing operation of the delay-time comparator for three states.

A thermostat must have hysteresis in the desired range to prevent output chatter and to control the temperature near a desired set-point temperature. In this study, a delay-time comparator with built-in custom hysteresis (also used in [6]) was adopted for both calibration and measurement modes to determine the lead or lag relationship between *t_D_* and *t_A_* (*t_C_*). Figure 13 shows a schematic diagram and the corresponding timing operation in three states. Two DFFs were used to sample the input signals *t_D_*, *t_A_*, and *t_A,shift_*, and a shift delay circuit was used to construct the detecting window (*i.e.*, hysteresis) based on *t_A_* and *t_A,shift_*. The width of the window was the hysteresis range. The delay circuit was realized using the AGTS to ensure that the design difficulty and circuit complexity did not increase greatly. If a resolution of 0.1 °C was designed and a 4 bit AGTS was used for achieving the adjustable shift delay, a maximal hysteresis with a range of approximately 1.5 °C can be achieved.

In the calibration mode, when *t_D_* leads or lags the detection window between *t_A_* and *t_A,shift_*, *Comp* is low (State A) or high (State B), respectively; subsequently, information on *Comp* is transmitted to the SAR logic to adjust the time gain appropriately. The output maintains its state when *t_D_* enters the window (State C), implying that the calibration has been completed and *m_i_* has been acquired. The operation in measurement mode is similar to that of calibration mode. The relationship between *t_D_* and the window is determined, and the output of the comparator is then sent to the thermal management system to control the temperature and avoid thermal damage.

## 3. Measurement Results

**Figure 14.**
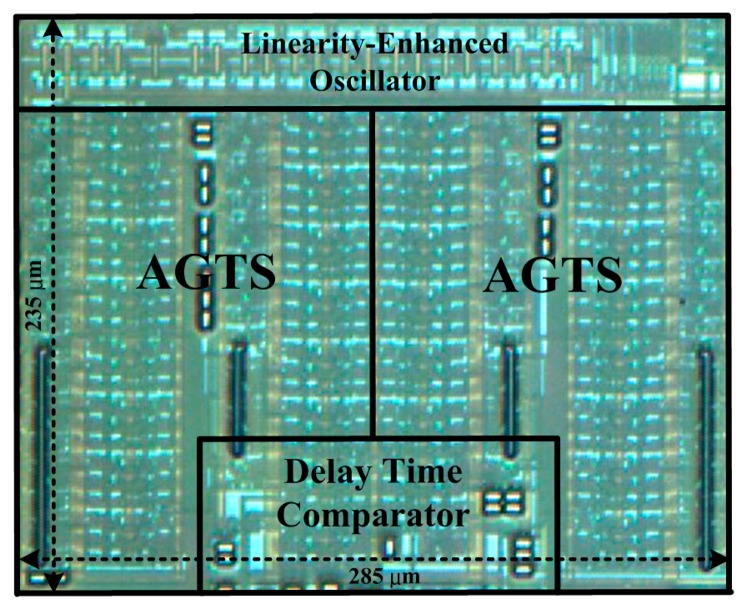
A floorplan of the proposed thermostat.

A floorplan of the proposed thermostat fabricated in a TSMC 0.35 μm CMOS process is shown in Figure 14; as shown in the figure, the core area is 0.067 mm^2^. Compared with the area of 0.12 mm^2^ occupied by a previous thermostat with two oscillators [6], an area reduction of approximately 40% was achieved by removing an oscillator and numerous bias circuits, and by using a more effective circuit layout. To minimize the effect of process variations and device mismatch, the two AGTSs were symmetrically arranged as close to each other as possible for delay matching. The test chips were placed in a chamber with programmable temperature and humidity (MHG-120AF) to evaluate the performance of the proposed linearity-enhanced technique. The Start signal and a reference frequency signal of 40 MHz (*i.e.*, oscillation period of 25 ns) were both generated using the FPGA board. An accurate digital oscilloscope (Agilent DSO7054A) was used to verify the timing of the measurement system.

**Figure 15.**
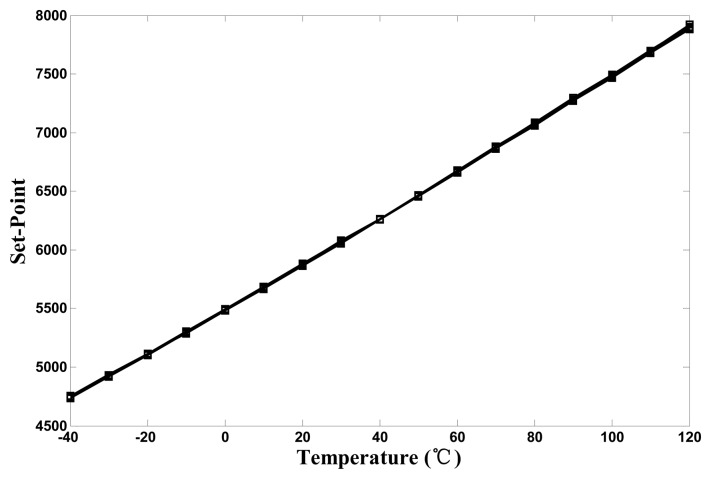
Trip temperature *versus* programmed set-point for eight chips.

In the calibration mode, TC was set to a median temperature of 40 °C from −40 to 120 °C, and NC was set to 6263. During the calibration procedure, the individual mi of the AGTS (*i.e.*, the time gain) in the temperature-sensing circuit could be obtained for each thermostat. In the measurement mode, the measurements were performed in 10 °C intervals over the −40 °C to 120 °C range. While increasing the test temperature, Ns in the timing-reference circuit was gradually varied from low to high to determine the circuit performance. Figure 15 shows the measurement results of eight chips. The set-point values of all chips were highly linear with the temperature and similar at any given trip temperature.

**Figure 16.**
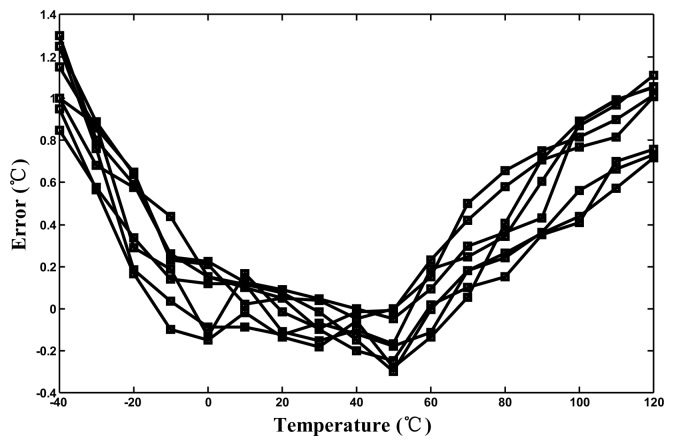
Corresponding errors after one-point calibration from −40 to 120 °C.

The effective resolutions were calculated to be approximately 0.05 °C. After one-point calibration, the corresponding inaccuracies derived from the measurement results ranged from −0.35 °C to 1.35 °C at operating temperatures between −40 °C and 120 °C, as shown in Figure 16. Compared with the maximum inaccuracy of approximately 6.0 °C for the un-linearized version, the linearity-enhanced thermostat subjected to process-variation calibration achieved an accuracy increase of more than threefold, validating that the linearity-enhanced technique functions successfully. For a single-supply voltage of 3.3 V, the measured power consumption was 25 μW at 10 Hz. The measured performance of the proposed thermostat and that of sensors proposed in other studies are presented in Table 1 for comparison. Compared this study with the related studies [5–9,12–18], the achieved accuracy after one-point calibration for a 160 °C operating range and the chip size with considering CMOS process are the best.

## 4. Conclusions

This paper presents a linearity-enhanced time-domain CMOS thermostat with process-variation calibration. The thermostat exhibited high accuracy, a wide operating temperature range, and one-point calibration support. Linearity enhancement was achieved using a compensated buffer and a simple buffer in parallel to form a new cell that generated a linearised delay PTAT. With the cell-based oscillator and the AGTS, the use of a delay-time comparator and SAR logic effectively eliminated the effect of process variations on temperature-sensing delay. In addition, a reference clock and another identical AGTS were used to program the set-point reference delay. The time comparison was achieved using the delay-time comparator with built-in custom hysteresis to generate the response to the calibration circuit or the thermal management system. The chip area of the proposed thermostat fabricated in a TSMC 0.35-μm CMOS process was only 0.067 mm^2^. Following one-point calibration, the maximal inaccuracy was improved substantially from 5.9 °C to 1.7 °C at operating temperatures of −40 °C to 120 °C, thus validating the combination of the proposed time-domain linearity-enhanced technique and calibration technique functions.

## Figures and Tables

**Table 1.** Performance comparison among related studies.

**Sensor**	**Resolution (°C)**	**Range (°C)**	**Error (°C)**	**Calibration**	**Power Consumption**	**Area (mm^2^)**	**CMOS Technology (μm)**
[5]	0.5	0∼75	±1.0	Two-Point	9 μW@20 Hz	0.4	0.35
[6]	0.05	0∼90	±0.6	Two-Point	30 μW@10 Hz	0.12	0.35
[7]	0.09	0∼90	±0.3	Two-Point	36.7 μW@2 Hz	0.6	0.35
[8]	0.045	−40∼120	−1.2∼0.2	Two-Point	23 μW@10 Hz	0.07	0.35
[9]	0.6	−40∼120	±1.1 ^#2^	Two-Point	0.09 μW@1 Hz	0.0036	0.13
[12]	0.139	0∼60	−5.1∼3.4	One-Point	150 μW@10k Hz	0.01	0.065
[13]	0.133	0∼100	−0.7∼0.6 ^#1^	One-Point	175 μW@1 kHz	NA	0.18/0.22
[14]	0.78	0∼100	±4	One-Point	1.2 mW@5 kHz	0.12	0.13
[15]	0.043	−40∼110	−2.8∼2.9	One-Point	400 μW@366 kHz	0.0066	0.065
[16]	0.595	20∼120	−0.6∼1.0 ^#1^	One-Point	289 μW@430 kHz	0.031	0.13
[17]	0.021	0∼130	−1.5∼1.3 ^#2^	One-Point	147 μW@400 kHz	0.0015	0.065
[18]	0.125	20∼75	±1.6	One-Point	7.88 μW@25 Hz	NA	0.065
This Work	0.05	−40∼120	−0.35∼1.35	One-Point	25 μW@10 Hz	0.067	0.35

#1 with off-chip second-order master curve fitting;

#2 simulated error.

## References

[1] Brokaw A.P. (1999). A Temperature Sensor with Single Resistor Set-Point Programming. IEEE J. Solid State Circuits.

[2] Microchip Inc. TC620 Series Logic Output Temperature Sensor. http://www.microchip.com.

[3] Lorenz P.S. (2005). Thermostat with Resistor-to-Digital-Converter Control of Trip Point.

[4] Duarte D.E., Geannopoulos G., Mughal U., Wong K.L., Taylor G. Temperature Sensor Design in a High Volume Manufacturing 65 nm CMOS Digital Process.

[5] Chen P., Chen T.-K., Wang Y.-S., Chen C.-C. (2009). A Time-Domain Sub-Micro Watt Temperature Sensor with Digital Set-Point Programming. IEEE Sens. J..

[6] Chen C.-C., Lin S.-H. (2013). A Time-Domain CMOS Oscillator-Based Thermostat with Digital Set-Point Programming. Sensors.

[7] Chen P., Chen C.-C., Peng Y.-H., Wang K.-M., Wang Y.-S. (2010). A Time-Domain SAR Smart Temperature Sensor with Curvature Compensation and a 3σ Inaccuracy of −0.4 °C∼+0.6 °C over a 0 °C to 90 °C Range. IEEE J. Solid State Circuits.

[8] Chen C.-C., Chen H.-W. (2013). A Linearization Time-Domain CMOS Smart Temperature Sensor Using a Curvature Compensation Oscillator. Sensors.

[9] Meng T., Xu C. (2009). A Cross-Coupled-Structure-Based Temperature Sensor with Reduced Process Variation Sensitivity. J. Semicond..

[10] Pertijs M.A.P., Bakker A., Huijsing J.H. A high-accuracy temperature sensor with second-order curvature correction and digital bus interface.

[11] Lin C.-W., Lin S.-F. (2012). A Linear CMOS Temperature Sensor with an Inaccuracy of ±0.15 °C. IEICE Electron. Express.

[12] Chung C.-C., Yang C.-R. (2011). An Autocalibrated All-Digital Temperature Sensor for On-Chip Thermal Monitoring. IEEE Trans. Circuit Syst. II.

[13] Chen P., Chen S.-C., Shen Y.-S., Peng Y.-J. (2011). All-Digital Time-Domain Smart Temperature Sensor with an Inter-Batch Inaccuracy of −0.7 °C ∼ +0.6 °C after One-Point Calibration. IEEE Trans. Circuit Syst. I.

[14] Ha D., Woo K., Meninger S., Xanthopoulos T., Crain E., Ham D. (2012). Time-Domain CMOS Temperature Sensors with Dual Delay-Locked Loops for Microprocessor Thermal Monitoring. IEEE Trans. VLSI Syst..

[15] Kim K., Lee H., Kim C. (2013). 366-Ks/s 1.09-nJ 0.0013-mm^2^ Frequency-to-Digital Converter Based CMOS Temperature Sensor Utilizing Multiphase Clock. IEEE Trans. VLSI Syst..

[16] An Y.-J., Ryu K., Jung D.-H., Woo S.-H., Jung S.-O. (2014). An Energy Efficient Time-Domain Temperature Sensor for Low-Power On-Chip Thermal Management. IEEE Sens. J..

[17] Chiang T.-T., Huang P.-T., Chuang C.-T., Chen K.-N., Chiou J.-C., Chen K.-H., Chiu C.-T., Tong C.-T., Hwang W. On-chip Self-Calibrated Process-Temperature Sensor for TSV 3D Integration.

[18] Xie S., Ng W.-T. A Low Power All-digital Self-Calibrated Temperature Sensor Using 65 nm FPGAs.

[19] Bharath K., Yao C., Kim C.-S., Ramanathan P., Saluja K.-K. A Low Cost Approach to Calibrate On-Chip Thermal Sensors.

[20] Lu S., Tessier R., Burleson W. (2014). Dynamic On-Chip Thermal Sensor Calibration Using Performance Counters. IEEE Trans. Comput. Aided Des..

[21] Demassa T.A., Ciccone Z. (1996). Digital Integrated Circuits.

[22] Filanovsky I.M., Allam A. (2001). Mutual Compensation of Mobility and Threshold Voltage Temperature Effects with Applications in CMOS Circuits. IEEE Trans. Circuits Syst. I.

